# The Growth of Medical Journalism

**Published:** 1894-07

**Authors:** 


### THE GROWTH OF MEDICAL JOURNALISM.

Forty-five years ago the leading medical journals published in the United States were the Boston *Medical and Surgical Journal*, the *American Journal of the Medical Sciences*, the *Southern Medical and Surgical Journal* (Augusta, Ga.), the *New Orleans Medical and Surgical Journal*, and the *St. Louis Medical and Surgical Journal*. These were representative of their sections, and were well conducted and patronized. There were about a dozen others. One of these was published in Philadelphia, one in New York, one in Buffalo, one in Columbus (Ohio), one in Cincinnati, one in Chicago, one in Louisville, one in Lexington (Kentucky), and one in Charleston (S. C.), etc. There was not a journal west of St. Louis. The organization of a medical journal in those days appears to have been no small undertaking. The autobiography of one of those in the above list states that it was "conceived in poverty and poverty finally brought it forth." We are glad to testify that this one, thus born in 1844, is now in vigorous health and one of our best exchanges.

A great change has come since that day, and now we have 234 medical journals published in the United States. Of these New York State has 52, Philadelphia alone has 20, Ohio has 14, and St. Louis, the city of medical colleges and proprietary medicines, has 12. West of St. Louis there are now 25. The enlargement of the scope of medicine and the development of specialism have been responsible for much of the increase. All of the fourteen journals published in 1850 were devoted to general medicine and surgery. Now, in addition to the large number of journals devoted to general medicine and surgery, we have three or four devoted to surgery alone; five to the eye, ear, nose and throat, six to obstetrics, gynecology and pediatrics, two to the skin and venereal diseases, ten to dietetics, hygiene and sanitation, one to the rectum, and finally there is one exponent of the new *fundamental* philosophy, orificial surgery, so-called. And we must not fail to mention those glittering publications which emanate from the offices of certain large drug houses under the name of medical literature. This keen division of medical journalism, it seems, is to be carried still further. We read of a journal just started called the *Refractionist*, and another to be published for the select use of colored physicians. The railroad surgeons now have their journal, and the "lady doctors" will have theirs. When we consider the immense progress that has been made of late years in certain departments of medicine, a certain amount of this specialism in modern journalism becomes proper enough, but it now appears to be reaching the limit of ridiculous excess. Railway surgery and the practice of medicine by female physicians, or colored physicians, are not special departments of medicine, and the establishment of periodicals under their auspices only increases the number of journals without improving the standard of journalism. We have too many journals. The profession could be served better with one-half the number, to say nothing of the happy effect which this reduction would have upon the status of medical journalism.

